# The Central Hospital Council for London

**Published:** 1913-01-18

**Authors:** 


### The Central Hospital Council for London.

A POLICY TOWARDS INSURED PATIENTS.

The following resolution was passed at the meeting of the Central Hospital Council held on January 7 at Westminster Hospital, viz.: "Resolved that the Central Hospital Council approves generally the system suggested by St. Bartholomew's Hospital for dealing with insured persons, leaving it to each individual hospital to make any minor modifications that experience may show to be necessary."

It will be remembered that the regulations adopted by St. Bartholomew's Hospital were as follows:

1. That all persons coming to the Hospital (except in cases of urgent illness) should be asked by a lay official of the Hospital whether they are insured.
2. That each insured person should be referred to a medical officer of the Hospital to decide whether his or her ailment be urgent or not.
3. That in the case of an insured person with an ailment or accident which can be treated by a general practitioner of ordinary competence, such insured person should be told by a medical officer of the Hospital that he or she must obtain treatment by a medical practitioner outside the Hospital.
4. That the treatment of the really necessitous poor remain as at present.

